# The Consequences of Internal Waves for Phytoplankton Focusing on the Distribution and Production of *Planktothrix rubescens*

**DOI:** 10.1371/journal.pone.0104359

**Published:** 2014-08-07

**Authors:** Peter Hingsamer, Frank Peeters, Hilmar Hofmann

**Affiliations:** Environmental Physics Group, Limnological Institute, University of Konstanz, Konstanz, Germany; University of Shiga Prefecture, Japan

## Abstract

Consequences of internal wave motion for phytoplankton and in particular for the distribution and production of the harmful and buoyant cyanobacterium *Planktothrix rubescens* were investigated based on data from two field campaigns conducted in Lake Ammer during summer 2009 and 2011. In both years, *P. rubescens* dominated the phytoplankton community and formed a deep chlorophyll maximum (DCM) in the metalimnion. Internal wave motions caused vertical displacement of *P. rubescens* of up to 6 m and 10 m, respectively. Vertical displacements of isotherms and of iso-concentration lines of *P. rubescens* from the same depth range coincided, suggesting that *P. rubescens* did not or could not regulate its buoyancy to prevent wave-induced vertical displacements. Diatoms dominated the phytoplankton community in the epilimnion and were vertically separated from *P. rubescens*. The thickness of the diatom layer, but not the diatom concentrations within the layer, changed in phase with the changes in the thickness of the epilimnion caused by internal wave motions. Seiche induced vertical displacements of *P. rubescens* caused fluctuations in the light intensity available at the depth of the *P. rubescens* layer. The interplay between seiche induced vertical displacements of the *P. rubescens* layer and the daily cycle of incident light lead to differences in the daily mean available light intensity between lake ends by up to a factor of ∼3. As a consequence, the daily mean specific oxygen production rate of *P. rubescens* differed by up to a factor of ∼7 between lake ends. The horizontal differences in the specific oxygen production rate of *P. rubescens* were persistent over several days suggesting that the associated production of *P. rubescens* biomass may lead to phytoplankton patchiness. The effect of internal seiches on the spatial heterogeneity and the persistence of horizontal differences in production, however, depend on the timing and the synchronization between internal wave motion and the daily course of incident light intensity. Vertical displacements caused by internal waves could be distinguished from other factors influencing the distribution of *P. rubescens* (e.g. active buoyancy control, production, vertical mixing) by a temperature-based data transformation. This technique may be of general use for separating wave-induced transport from other processes (e.g. sedimentation, vertical mixing) that affect the distributions of dissolved substances and suspended particles.

## Introduction

Lakes are dynamic systems and organisms living within lakes are influenced by physical, chemical and biological factors which vary at different temporal and spatial scales. The relative importance of these factors for the development and distribution of organisms and populations depends on the temporal-spatial scales in relation to e.g., size, growth rate, and mobility of the organism [1], [2].

The vertical distribution of phytoplankton in the water column is a consequence of nutrient and light availability, competition, and their sedimentation rate or ability to control buoyancy [3]. Vertical and horizontal currents have a strong influence on the distribution of organisms as they can induce passive transport or changes in behaviour, depending on the swimming ability of the organism and the background current field [4].

One of the main causes of currents below the surface water layer in stratified aquatic systems is internal waves. Internal waves are common in deep lakes and typically have maximum amplitudes in the metalimnion. Basin-scale internal oscillations, which can be understood as a superposition of propagating internal waves, occur in almost all lakes and are called internal seiches. Seiches are typically generated by wind forcing. The fundamental mode basin-scale internal seiche is characterized by a periodic and predominantly horizontal flow of water in epilimnion and hypolimnion. The water flows in opposite directions in the two layers such that the net water column flow is zero everywhere in the basin. The flow pattern results in an oscillation of the thermocline with opposite vertical displacements of the thermocline at the two ends of the basin [5]. The transport of water masses associated with seiching results in horizontal and vertical displacement of organisms, whereby the vertical displacements are maximal at the ends of the lake and the horizontal displacements are largest in the middle of the lake. Internal wave motions influence the distribution of species of different trophic levels and sizes by causing passive transport or by inducing an active response of the organisms. The effects of internal waves on the distribution of organisms in lakes have been described for phyto- and zooplankton [6]–[8] and for fish [9]. Internal waves can enhance the availability of nutrients in the metalimnion by energy dissipation in the lake boundary layer or by mixing due to overturns in the interior of the lake [10]–[12]. As internal waves change the vertical position of organisms and therefore the amount of available light, Cuypers et al. [6] suggested, that they can influence the growth rate of *Planktothrix rubescens* (former *Oscillatoria rubescens)*. Garneau et al. [13] presented field data showing the effect of short term displacements on the growth rate of *P. rubescens.* They found that vertical displacements of *P. rubescens* over a time period of three days altered available light sufficiently to cause a significant impact on production of *P. rubescens* [13].

*P. rubescens* is an ubiquitous, potentially toxic, and filamentous cyanobacteria species which is able to control its buoyancy using gas-filled vesicles [14]. *P. rubescens* typically forms a deep chlorophyll maximum (DCM) in the metalimnion of lakes during the stratified season and has been reported in lakes all over the world with different lake area and depth [6], [15]–[19]. The steep metalimnetic temperature gradient, which typically corresponds to a steep density gradient in freshwater lakes, gives *P. rubescens* the ability to persist against vertical mixing [20]. In Lake Ammer, our study site, a toxic strain of *P. rubescens* regularly forms blooms with a potentially negative effect on whitefish, an important species for local commercial and recreational fishery [21], [22]. Halstvedt et al. [23] found that toxin concentration in the water depends mainly on the biomass of *Planktothrix spp.* Therefore, detailed knowledge about *P. rubescens* abundance and distribution is of significant importance for water treatment, fisheries, and tourism [21], [24].

Environmental conditions required for the formation of cyanobacteria blooms in general, and for *P. rubescens* in particular, are still not completely understood. Eutrophication and climate change seem to play an important role. However, blooms of *P. rubescens* were often associated with re-oligotrophication [15], [25], [26] and earlier stratification of the water column [16], [27]. Posch et al. [28] concluded that strong stratification and changes of nutrient content to higher N to P ratios favour *P. rubescens*.

This study investigates the impact of internal wave motions on the vertical distribution of phytoplankton in Lake Ammer with particular focus on the consequences of internal waves on the vertical distribution and production of *P. rubescens*. The investigations combine the analysis of the consequences of physical processes affecting vertical distribution patterns of *P. rubescens* with production calculations. Furthermore, the study also considers different phytoplankton taxa, buoyant *P. rubescens* and diatoms. Based on field observations from Lake Ammer, we characterise the internal wave field and the vertical distributions of diatoms and *P. rubescens* and the temporal changes in these distributions. The vertical displacements of *P. rubescens* are compared to isotherm displacements indicating internal wave motions. We test whether wave-induced displacements have a significant influence on the production of *P. rubescens* and whether they may cause spatial differences in production over short- and long-term periods. We further investigate how diatom distributions are affected by internal waves. Finally, we present a method to distinguish advective motions due to internal wave motions from other changes in the spatial distribution of *P. rubescens*, neutrally buoyant particles or dissolved substances, and show the limits of the buoyancy regulation of *P. rubescens*.

## Materials and Methods

### Study site

**Figure 1:**
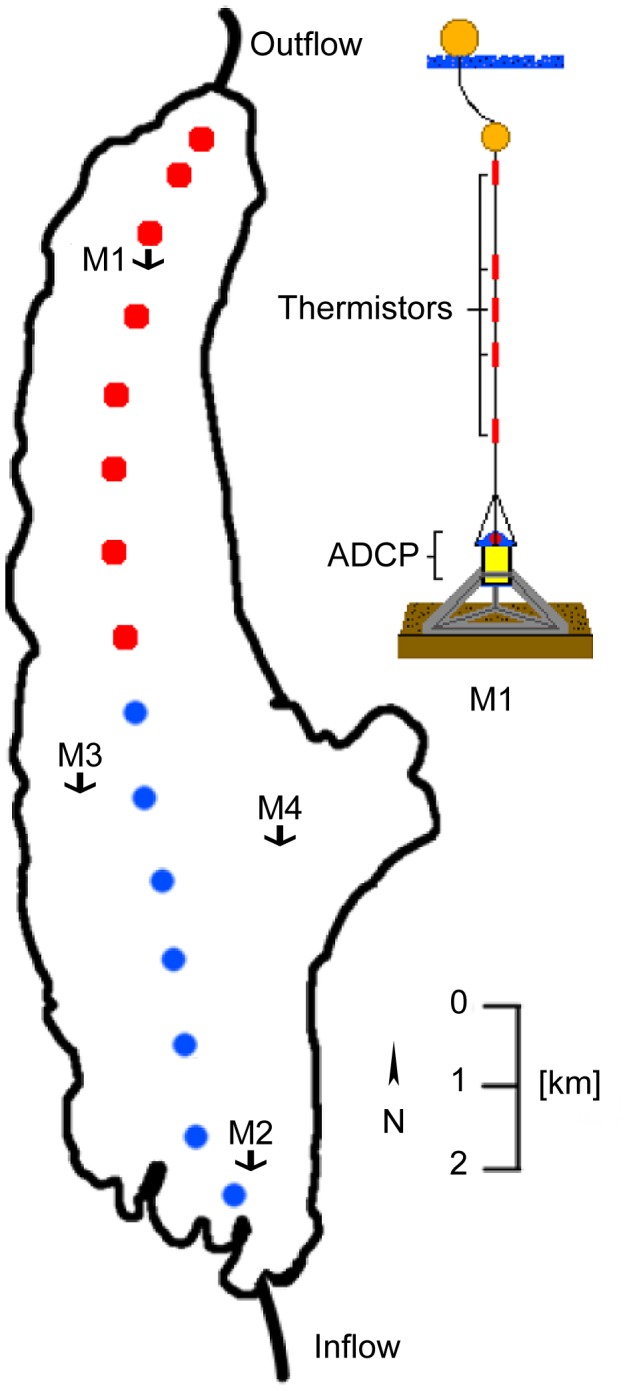
Overview on the measuring stations on Lake Ammer. In 2009 CTD and MFP profiles were collected along a north-south transect at all stations indicated by dots (transect T1). In 2011 only the stations marked in red (transect T2) were sampled repeatedly during 34-hour period. In 2011 thermistor chains were moored at stations M1 to M4. An ADCP was also deployed at station M1. The right drawing shows a schematic of the ADCP and thermistor chain mooring at M1.

Field measurements were conducted in Lake Ammer (Fig. 1, with permission of the Bavarian Lake Administration and the District Office Landsberg-Lech), a prealpine lake in the South of Germany (altitude: 553 m; coordinates 47°59′ N, 11°07′ E). It has a surface area of 46.6 km^2^, a catchment area of 993 km^2^, and a volume of 1.7 km^3^. The maximum and mean depths are 81.1 m and 37.5 m, respectively. Lake Ammer is a dimictic lake with a water residence time of about 2.7 years. The main axis of Lake Ammer has a length of 16 km and is oriented in a north-south direction. The lake width is about 2–3 km and reaches 5 km at the Bay of Herrsching. During our study period the mean depth of the surface mixed layer (*z_mix_*) was∼8 m and ∼6 m in 2009 and 2011, respectively (see below for definition of *z_mix_*). Lake Ammer was eutrophic in the 1970s. Since the early 1980s, phosphor levels declined in the process of re-oligotrophication such that the trophic state of the lake is mesotrophic nowadays [29], [30]. Mean total phosphorous concentrations in the entire water column were ∼7 µg L^−1^ and ∼7.5 µg L^−1^ and in the upper 20 m 9 µg L^−1^ and 10 µg L^−1^ in summer 2009 and 2011, respectively (data provided by the Bavarian Environmental Agency).

### Field campaigns, design of the experiments and instrumentation

Two field campaigns were conducted on Lake Ammer, one in the summer of 2009 and one in the summer of 2011, respectively. The field study did not involve endangered or protected species and did not take place on protected land. On the 24^th^ of August 2009, 16 vertical profiles of the phytoplankton assemblage and abundance, and of abiotic parameters were collected along a north-south transect which had a horizontal spacing of 1 km between the sampling stations (Fig. 1, dots). In 2011, between 13^th^ and 14^th^ of August, vertical profiles of similar parameters as in 2009 were measured repeatedly over a period of 34 hours along a 7 km long transect T2 in the northern part of the lake (Fig. 1, red dots). For each transect data collection required approximately 2 hours and a total of 10 transects were measured: A: 9∶00 (8∶00–10∶00), B: 13∶00 (12∶00–14∶05), C: 16∶00 (15∶00–16∶55), D: 20∶00 (18∶45–20∶30), E: 22∶00 (21∶10–23∶00), F: 2∶00 (1∶20–3∶00), G: 4∶00 (3∶35–5∶15), H: 8∶00 (7∶20–9∶20), I: 11∶00 (10∶00–11∶50) and J: 17∶00 (16∶30–18∶15). As in 2009, the spatial distance between profiling stations was ∼1 km. During both field campaigns all vertical profiles covered a depth range from the water surface down to 30 m or to the lake bottom.

The vertical profiles of phytoplankton assemblage and abundance were measured with a multi-spectral FluoroProbe (Moldaenke FluoroProbe, MFP). The MFP measures fluorescence at different wavelengths (470, 525, 570, 590, and 610 nm) and utilises spectral information to discriminate between different phytoplankton groups. In the standard configuration, the Moldaenke software converts the recorded fluorescence values into chlorophyll-*a* equivalent (Chl-*a* eq.) concentrations of green algae, diatoms, cryptophytes, and cyanobacteria [31].

The fluorescence signals of *P. rubescens* correspond well to the factory-calibrated fingerprint of cryptophytes, as is indicated by lab experiments with *P. rubescens* isolates. Cryptophytes have a low abundance in Lake Ammer. Chl-*a* eq. concentrations assigned by the MFP to cryptophytes are therefore rather a measure of the abundance of *P. rubescens* [32]. This has been confirmed by a comparison of data from the MFP with data on the abundance of *P. rubescens* and other phytoplankton obtained by cell counting in water samples which were collected during the two field campaigns [32]. We therefore use the MFP output for the cryptophyte channel as the Chl-*a* eq. concentration of *P. rubescens*. In the vertical profiling the MFP was lowered with a speed of approximately 5 m min^−1^ and measured at a sampling rate of 1 Hz thus providing a vertical resolution of 5–15 cm.

Vertical profiles of temperature and underwater light intensity were measured with a CTD probe (RBR Ltd., Canada) which was equipped with a spherical light sensor (LI-COR, USA). The CTD-probe sampled at 6 Hz and was lowered together with the MFP.

Only data from the downcast of the profiling instruments were considered in the further analysis. All profiles from the MFP and the CTD-probe were linearly interpolated to obtain regularly-spaced data sets in the top 30 meters at 0.1 m depth intervals.

During the field campaign in 2011, moorings were installed at stations M1, M2, M3, and M4 (Fig. 1). All moorings were equipped with temperature loggers (TR-1060, RBR Ltd., Canada; accuracy ±0.002°C) measuring at a sampling interval of 5 s between the 1^st^ of July 2011 and 21^st^ of September 2011. The temperature loggers were mounted with a 2 m vertical spacing from 1 m down to 7 m water depth, 1 m spacing between 7 m and 15 m water depth, again 2 m spacing between 15 m and 25 m and thereafter 5 m spacing down to the bottom (50 m). At the bottom of station M1 an acoustic Doppler current profiler (600 kHz ADCP; RDI Workhorse Sentinel; Teledyne RD Instruments, USA) was installed (Fig. 1) measuring upwards through the water column with a vertical resolution of 0.5 m. The ADCP averaged internally 20 individual profiles taken every 1.5 s, thus providing a sampling interval of 30 s.

### Data analysis

#### Layer definitions

The Chl-*a* eq. concentration profiles of *P. rubescens* indicate a distinct depth region of increased abundance of *P. rubescens*. In the following, the depth range in which Chl-*a* eq. concentrations of *P. rubescens* exceed 8 µg L^−1^ is identified as “*P. rubescens* layer”. The layer limit of 8 µg L^−1^ Chl-*a* eq. *P. rubescens* corresponds to about 70% of the mean of the maximum concentrations of *P. rubescens* in the profiles from the field campaign in 2009 (11.1 µg L^−1^ Chl-*a* eq. *P. rubescens*) and in the profiles from the field campaign in 2011 (11.4 µg L^−1^ Chl-*a* eq. *P. rubescens*). The “depth of the *P. rubescens* layer” is defined as the depth at which the *P. rubescens* concentration has a maximum within a vertical profile.

The boundaries of the diatom layer were defined as 70% of the mean maximum concentration, leading to a layer limit of 1.4 µg L^−1^ Chl-*a* eq. and 1.6 µg L^−1^ Chl-*a* eq. for 2009 and 2011, respectively.

The surface mixed layer depth was calculated from the temperature profiles as the depth at which the density was 0.1 kg m^−3^ larger than the density at 2 m water depth [33]. The metalimnion was defined as the layer that extends from below the mixed layer depth to the depth at which the density gradient decreases to 0.05 kg m^−4^. The density gradient chosen to indicate the lower limit of the metalimnion is about 75% of the average maximum density gradient in the thermocline. Additionally, it corresponds to the minimum density gradient that allows *P. rubescens* to stay in a distinct water layer [34]. The hypolimnion is the water body below the metalimnion.

#### Spectral analysis

Time series of the depth of different isotherms were calculated from the thermistor-chain data. Spectral analysis was applied to the time series of the depth of the 10°C isotherms. Power spectra and 95% confidence intervals were calculated using half-overlapping windows (segments) and employing the Welch-method with a Hamming window. The confidence interval becomes smaller the more windows are available for averaging, but the maximum period which can be resolved in the spectrum decreases with decreasing window size. As we intended to resolve the spectrum at a wide range of periods, we applied several window sizes (10 days, 5 days, 8 hours, and 1 hour) to improve the confidence interval for the spectral power at short time periods.

#### Underwater light availability and oxygen production of *P. rubescens*

Profiles of photosynthetic active radiation (PAR, 400–700 nm) and their temporal variation are calculated from the Lambert-Beers law using the temporal course of incident PAR at the lake surface and a light extinction coefficient determined from the profiles of PAR measured with our PAR sensor connected to the CTD. Incident PAR at the lake surface is determined from data on global radiation (W m^−2^) with a temporal resolution of one hour. Data on global radiation were available for early August and for September (DWD weather station Hohenpeiβenberg). The conversion factors of Tsubo and Walker [35] and Wetzel [36] (1 W m^−2^ = 2.116 µmol photons m^−2^ s^−1^ PAR) are used to estimate PAR from the radiation data. In parts of the analysis we have used as incident light intensity clear sky radiation also provided by DWD weather station Hohenpeiβenberg.

The light extinction coefficient in the water column is determined from linear regression of the log transformed underwater PAR versus depth. Only data from the depth range between 1.5 m and 10 m were considered, as the PAR near the surface may be affected by shading effects of the boat and PAR data from below 10 m depth were rather low and therefore had a high noise level. Combining the time series of incident PAR with the extinction coefficient for PAR provides an hourly resolution of the temporal changes in the vertical profiles of PAR. Thus, PAR available for *P. rubescens* can readily be determined from the vertical position of *P. rubescens*. *P. rubescens* production is calculated using the P/I relationship from Walsby et al. [37] which describes oxygen production by *P. rubescens* in the absence of nutrient limitation:
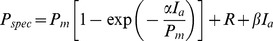
(1)




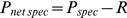
(2)




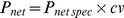
(3)where *P_spec_* (eq. 1) is the specific O_2_ production rate, and *I_a_* the light intensity available at the depth of the *P. rubescens* layer. *P_net spec_* is the net specific O_2_ production rate (eq. 2), *P_net_* the net production rate per unit volume (eq. 3) and *C_V_* the concentration of cell volume of *P. rubescens*. Photosynthetic coefficients (*P_m_, α, β, R*) were taken from Walsby et al. [37]. The concentration of cell volume of *P. rubescens* was calculated using the data from the MFP, the conversion factors from Hofmann and Peeters [32] for cell abundance (1 µg Chl-*a eq* L^−1^ = 2,400 cells mL^−1^) and the typical biovolume of *P. rubescens* cells in Lake Ammer (1 cell = 76.8 µm^3^) given by Ernst et al. [22].

Analysis and graphs were done using MatLab 2013b (MathWorks Inc., 2013).

## Results

### Internal wave field

**Figure 2:**
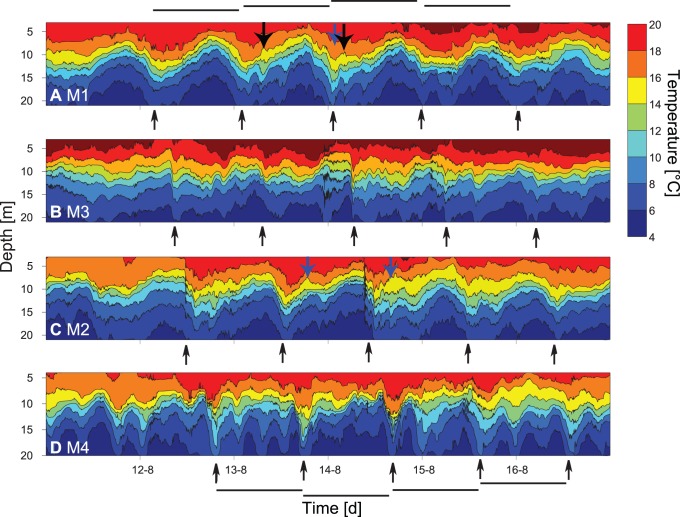
Temporal course of temperature isolines at moorings M1–M4 in the uppermost 20 m of the water column. Black arrows below each panel mark the inflection points of temperature isolines due to the longitudinal basin-scale internal wave. Vertical mode 2 internal waves are indicated by solid arrows within the figure. Graphs are ordered in the expected sequence of the Kelvin wave starting in the north with M1 followed by M3, M2 and M4 (see also Fig. 1). The length of the black lines represents time periods of ∼23 hours, the expected period of the longitudinal basin-scale wave.

**Figure 3:**
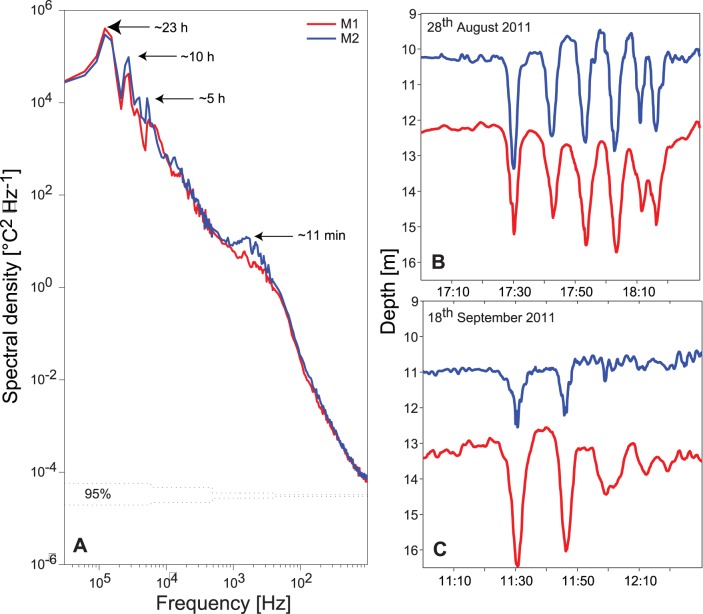
Spectra of the 10°C isotherm and examples high frequency internal waves trains. A: Spectrum of the time series of the 10°C isotherm depths at stations M1 (red) and M2 (blue) from 20^th^ of August to 20^th^ of September 2011. B: and C: examples of trains of high frequency internal waves t in Lake Ammer shown as isotherm displacements of the 10°C (blue) and 8°C isotherm (red).

Data from all thermistor chains deployed in 2011 show periodic fluctuations of isotherm depths (Fig. 2) which indicate the presence of internal waves. At the northernmost and southernmost stations, M1 and M2, the isotherm fluctuations have particularly large amplitudes and occur regularly with a period of ∼23 hours (arrows below the panels, Fig. 2). The spectra of the 10°C isotherms measured at M1 and M2 between the 20^th^ of August and 20^th^ of September confirm that the time series of the isotherm fluctuations from the two stations both have a spectral peak at ∼23 hours (Fig. 3A), are coherent and are phase shifted by ∼170°. These results suggest that the isotherm fluctuations with a ∼23 hour period are caused by a vertical mode 1 and horizontal mode 1 (V1H1) longitudinal internal seiche (Figs. 2 and 3). This is further supported by the calculation of the phase speed of a vertical mode 1 internal wave propagating in a fluid with the mean background stratification of Lake Ammer during the evaluation period. Based on the density profile, determined from the average temperature profile measured by the thermistor chain at station M1 and the mean basin depth of ∼40 m, a phase speed of 0.26 m s^−1^ was obtained for the first vertical mode wave. The basin length in north-south direction is ∼10 km at the 40 m depth isocline. As the V1H1 seiche in a rectangular basin has a wave length of twice the basin length, the period of the fundamental mode standing wave with a phase speed of 0.26 m s^−1^ becomes ∼22 hours, which supports the hypothesis that the observed oscillations with a ∼23 hours period are due to a longitudinal basin-scale V1H1 internal seiche. As the stratification in 2009 and 2011 was similar, the seiche can be expected to have about the same period in both years.

A more detailed analysis of the data from the four moorings reveals that the maximum downward deflection of the isotherms passes the different stations in a regular repeated order starting at station M1 and continuing to stations M3, M2 to M4 (arrows below panels in Fig. 2, stations are depicted in Fig. 1). This pattern indicates a counter-clockwise propagation of the internal front suggesting that the basin-scale internal wave is affected by the Coriolis force and can be considered as a Kelvin-type internal wave.

In addition to the peak at ∼23 hours the spectral analysis of the isotherm fluctuations shows peaks at ∼10 and ∼5 hours, and a broad peak at ∼11 minutes (Fig. 3A). The isotherm fluctuations with a 10-hour period at stations M1 and M2 are coherent and in phase suggesting that they result from a horizontal mode 2 vertical mode 1 basin-scale seiche (V1H2) oscillating in a north-south direction. The 5-hour peak may result from a cross-basin V1H1 seiche with oscillating water movements in an east-west direction. The increase in variance at ∼11 minutes is most likely related to high frequency internal waves, which were observed several times at station M1. Examples of such waves, observed e.g. on the 28^th^ of August and on the 18^th^ of September, are shown in Fig. 3B and C. The observed periods of the high frequency internal waves are clearly separated from the period of the basin-scale surface seiche of Lake Ammer of ∼28 minutes as estimated from Merian’s formula [36]. The shape of the observed vertical fluctuations of the isotherms at time scales of several minutes are typical for trains of high frequency solitary waves (Fig. 3B and C). These features were observed in consecutive order at the different stations in Lake Ammer, suggesting that trains of solitary waves propagate regularly along the main axis of the lake in close connection with the internal front associated with the basin-scale Kelvin wave.

**Figure 4:**
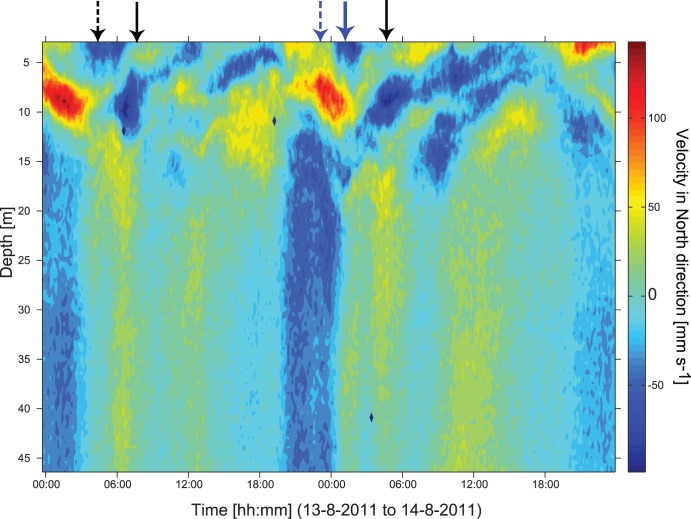
Current velocity (north component) at station M1 during the 34 hour campaign in August 2011. Arrows indicate specific current profiles related to mode 2 internal waves and preceding internal fronts: A three layer current profile with southward flow in epi- and hypolimnion and northward flow in the metalimnion (blue solid arrow), flow associated with a preceding front propagating towards shore (blue dashed arrow) and a three layer current profile (black solid arrow) and a preceding internal front (black dashed arrow). with flow in opposite direction.

**Figure 5:**
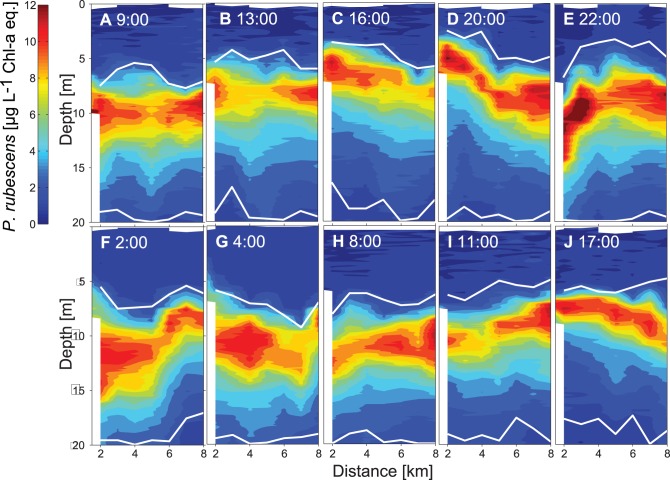
Spatiotemporal course of the *P. rubescens* layer (µg L^−1^ Chl-*a* eq.) measured along the transect T2 between the 13^th^ and 14^th^ of August 2011. The mooring M1 (thermistor chain and ADCP) was deployed 2.5 km from the north. White lines mark the bottom boundary of the upper mixed layer and the upper boundary of the hypolimnion, respectively.

Beside vertical displacements of the thermocline that are typical for vertical mode 1 internal waves, the thickness of the metalimnion changes during certain times, e.g. at station M1 on 13^th^ of August between 6∶30–9∶00 (Fig. 2A, black arrow within panel) and on 14^th^ of August between 0∶45 and 2∶30 (Fig. 2A, blue arrow within panel). The vertical spreading of the metalimnion is associated with a three-layered current field. On 14^th^ of August this current field is characterised by a southward-directed current in the epilimnion, a northward-directed current in the metalimnion, and a southward-directed current in the hypolimnion (Fig. 4, blue arrow). The structure of the current field and the vertical spreading of the isotherms are typical for vertical mode 2 internal waves, and apparently occurred shortly after the internal front associated with the basin-scale wave has propagated towards the shore (23∶00 on 13^th^ of August; Fig. 4, blue dashed arrow). The passage of the internal front is indicated by high currents in the epilimnion, a rapid downward displacement of the isotherms and an increase in epilimnion thickness with time. Note, that this sequence of events occurs just between the measurements of the transects from 22∶00 on 13^th^ and 2∶00 on the 14^th^ of August (Fig. 5E and F). The change in the thermal structure between these transects, i.e. the downward displacement of the thermocline at the northern end and the spreading of isotherms in the metalimnion apparently result from the internal front and the vertical mode 2 internal wave. We did not observe any inverse temperature stratification at thermistor chain M1 that would be typical for internal wave breaking associated with mixing during 13^th^ of August and 14^th^ of August.

At 8∶00 on the 13^th^ of August the vertical mode 2 wave was associated with a three-layered current field opposite to that at 2∶00 on the 14^th^ of August (Fig. 4, blue arrow). The vertical mode 2 wave from 8∶00 on the 13^th^ of August occurred shortly after the passage of an internal front that propagated southwards away from shore as is indicated by the strong southward current in the epilimnion (Fig. 4, dashed black arrow). Another similar vertical mode 2 wave took place on the 14^th^ of August at 4∶30, which had the same current structure as the vertical mode 2 wave described for 13^th^ of August (Fig. 4, black arrow). Vertical mode 2 waves also occurred at the opposite end of the basin at station M2 shortly after the passage of the internal front, e.g. at 18∶30 on 13^th^ and at 16∶00 on the 14^th^ of August (Fig. 2C, blue arrows within panel).

### Spatial distributions of Chl. a, *P. rubescens*, diatoms and temperature

In 2009 as well as in 2011, total Chl-*a* eq. concentrations reached maximum concentrations (∼14.3 µg L^−1^ Chl-*a* eq. and ∼14.6 µg L^−1^ Chl-*a* eq., respectively) in the upper metalimnion indicating the existence of a pronounced deep chlorophyll maximum (DCM). *P. rubescens* dominated the phytoplankton community in Lake Ammer reaching maximal abundances in the DCM of ∼33,000 cells mL^−1^, corresponding to ∼14.6 µg L^−1^ Chl-*a* eq. *P. rubescens*. The mean of the Chl-*a* eq. maximum concentrations of *P. rubescens* in the profiles from 2009 and 2011 were 11.1 µg L^−1^ (standard deviation 1.7 µg L^−1^) and 11.4 µg L^−1^, (standard deviation: 0.8 µg L^−1^), respectively, and thus did not significantly differ between the two years (two-sided t-test: p = 0.62).

In both years, diatoms were the second most abundant phytoplankton group and dominated the phytoplankton community in the epilimnion. Diatoms formed an upper phytoplankton layer and reached maximum concentrations of about 3.5 µg L^−1^ Chl-*a* eq. The mean maximum Chl-*a* eq. concentration of diatoms in the profiles from 2009 (1.91 µg L^−1^) was significantly lower than in the profiles from 2011 (2.30 µg L^−1^) (two-sided t-test: p = 0.02).

**Figure 6:**
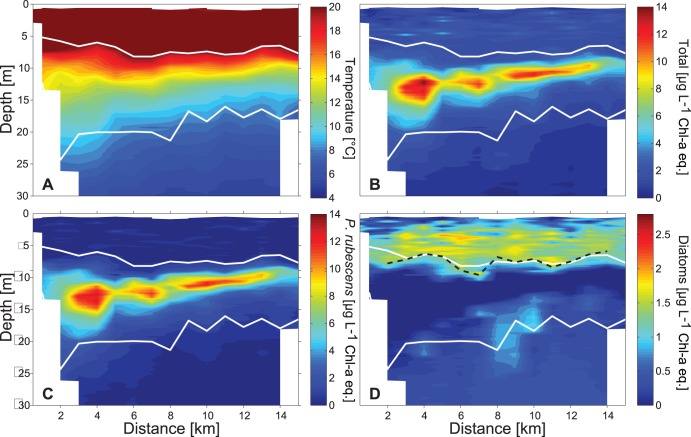
Temperature, T-Chl-*a* eq., *P. rubescens* and diatoms along the north-south transect. Spatial distribution of A: temperature (°C), B: T-Chl-*a* eq. (µg L^−1^), C: *P. rubescens* (µg L^−1^ Chl-*a* eq.), and D: Diatoms (µg L^−1^ Chl-*a* eq.) along the north-south transect (T1) on 24^th^ of August 2009. Distance is measured from the northern shore. White lines indicate the depth of the upper mixed layer and the boundary between metalimnion and hypolimnion. The black-dashed line in D marks the lower limits of the diatom layer below which the diatom concentration is less than 1.4 µg L^−1^ Chl-*a* eq.

**Figure 7:**
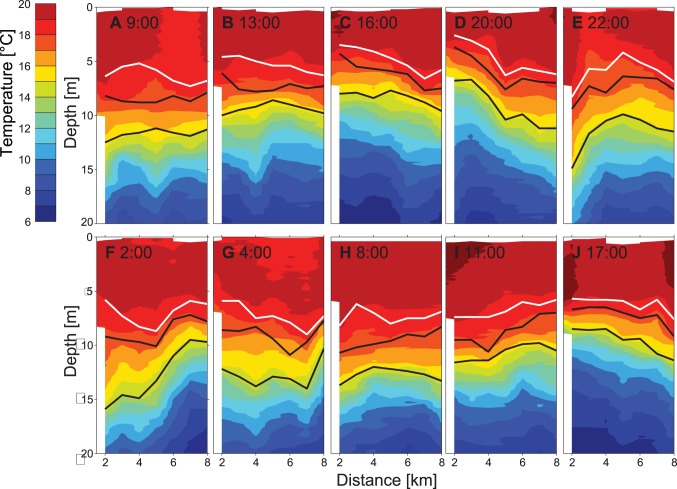
Spatiotemporal course of temperature (°C) measured along the transect T2 between the 13^th^ and 14^th^ of August 2011. The mooring M1 (thermistor chain and ADCP) was deployed at 2.5 km distance from the north. The black lines mark the upper and lower limits of the *P. rubescens* layer (concentration ≥8 µg L^−1^ Chl-*a* eq.). The white lines mark the lower limits of the diatom layer below which the diatom concentration is less than 1.6 µg L^−1^ Chl-*a* eq.

In 2009, the mean depths of the upper boundary of the metalimnion and of the *P. rubescens* layer were ∼2 m larger than in 2011 (mean mixed layer depth 2009: ∼8 m and 2011 ∼6 m; Figs. 6A and C and 5 and 7). Consistently, the thickness of the epilimnion was larger in 2009 than in 2011 and the diatoms distributed within the epilimnion extended over a larger depth range in 2009. The temperature at the depth of the *P. rubescens* layer ranged between 11.5°C and 14.4°C in 2009 and between 14.0°C and 18.5°C in 2011.

In 2009 the distributions of *P. rubescens*, total Chl-*a* eq. and temperature indicate that the depth of the *P. rubescens* layer, the depth of the maximum total Chl-*a* eq. and the depths of the isotherms close to the *P. rubescens* layer decrease substantially from north to south by about 5 m (Fig. 6). The tilts of the *P. rubescens* and total Chl-*a* eq. layers agree well with the tilt of the isotherms. The thickness of the *P. rubescens* layer, of the total Chl-*a* eq. layer and of the metalimnion decreases from north to south. The concentrations of *P. rubescens* within the *P. rubescens* layer did not change continuously but showed a patchy distribution along the north-south transect. Nevertheless, because of the larger thickness of the *P. rubescens* layer in the north the total abundance of *P. rubescens* within the top 30 m of the water column is substantially larger in the northern part than in the southern part of the lake. The *P. rubescens* abundance integrated over the upper 30 m is well correlated with the thickness of the metalimnion (R = 0.66, p = 0.02).

Although the thickness of the metalimnion and of the *P. rubescens* layer decreased from north to south, the surface mixed layer depth and hence the vertical extent of the epilimnion remained essentially constant (Fig. 6A and D, upper white lines). Also the thickness of the diatom layer did not substantially change along the north-south transect (Fig. 6D, dashed black line).

**Figure 8:**
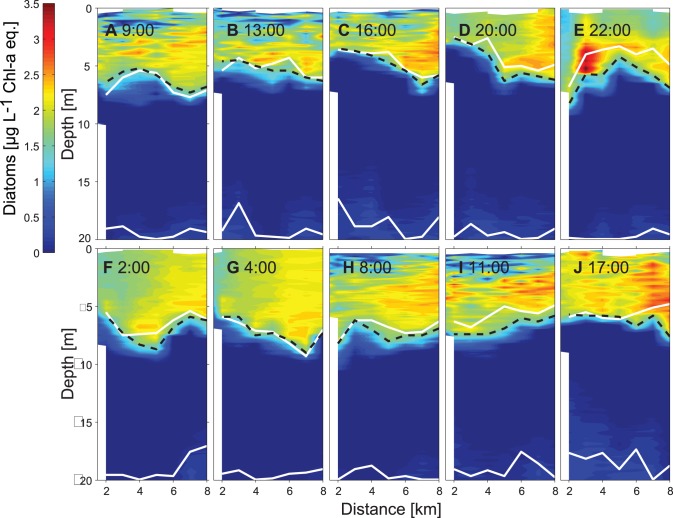
Spatiotemporal course of diatoms (µg L^−1^ Chl-*a* eq.) measured along the transect T2 between the 13^th^ and 14^th^ of August 2011. White lines mark the bottom boundary of the upper mixed layer and the upper boundary of the hypolimnion, respectively. The black-dashed lines marks the lower limits of the diatom layer below which the diatom concentration is less than 1.6 µg L^−1^ Chl-*a* eq.

In 2011 the distributions of *P. rubescens*, diatoms and temperature were measured repeatedly over 34 hours (from 8∶00 on the 13^th^ of August to 18∶00 on the 14^th^ of August) along transect T2. The vertical and horizontal structure of the distributions of the three variables changed rapidly in time. However, several properties of the distributions show the same vertical and horizontal pattern in all transects (Figs. 5, 7–8). In particular the lower limit of the diatom layer and the mixed layer depth, as well as the depth of the *P. rubescens* layer and the depth of the 16.5°C isotherm are correlated (R = 0.84, p<0.001 and R = 0.87, p<0.001, respectively). As in 2009, *P. rubescens* biomass integrated over the upper 30 m was correlated with the thickness of the metalimnion (R = 0.56 p<0.001).

The transect average of the depth of the upper limit of the *P. rubescens* layer decreases in the first half of the 13^th^ of August until about 20∶00 and increases over the night reaching a maximum at 22∶00. Afterwards the transect averaged layer depth decreases again in a similar fashion as on the 13^th^ of August, becomes maximal at 8∶00 on 14^th^ of August and increases thereafter as on the previous day.

At the beginning of the campaign the *P. rubescens* layer is essentially horizontal and located between 8 and 12 m water depth (Fig. 5A), which corresponds to the depth range between the 15.0°C and 17.4°C isotherm (Fig. 7A). In the transects between 16∶00 and 22∶00 on 13^th^ of August, isotherms and the *P. rubescens* layer are tilted upwards towards the northern end of the transects (Figs. 5C and D and 7C and D). Thereafter, isotherms and the *P. rubescens* layer were rapidly deflected downwards (Figs. 5E and 7E) followed by an increase in the thickness of the *P. rubescens* layer of ∼2–3 m at the northern end of the transect (Figs. 5F and 7F). The timing of the rapid deflection of the layer corresponds with the time of the passage of the internal front. The timing of the vertical spreading of the layer coincides with the time of the passage of the vertical mode 2 internal wave (Figs. 2 and 4). Along all transects the variations in the depth of the *P. rubescens* layer and in the depths of the isotherms in the metalimnion are very similar, as illustrated by the close agreement between the spatial variation of the 15°C and 17°C isotherms and the iso-concentration lines indicating 8 µg L^−1^ Chl-*a* eq. of *P. rubescens* (Fig. 7). Considering all profiles measured during the 34-hour campaign in 2011, the temperature at the depth of the *P. rubescens* layer is on average 16.4°C. The close association between the depth of the *P. rubescens* layer and the depth of the 16.4°C isotherm in each of the transects (Figs. 5 and 7) suggests that the same processes are responsible for the major part of the vertical movements of isotherm and *P. rubescens* layer depths.

Diatoms are most abundant in the epilimnion and are vertically separated from the *P. rubescens* layer (Fig. 8). The depth of the lower boundary of the diatom layer changes in parallel to the depth of the surface mixed layer (Fig. 8, upper white line and dashed black line). Because vertical displacements of the mixed layer depth cause temporal variations in the thickness of the epilimnion, the vertical extension of the diatom layer changes accordingly (Fig. 8). The diatom concentration in the epilimnion however remained rather unchanged and thus diatom concentration and epilimnion thickness are not correlated (R = 0.12, p = 0.34). Hence, diatom biomass integrated over the upper 30 m is very well correlated with the thickness of the epilimnion (R = 0.70 p<0.001).

### Underwater light climate and production

Light extinction coefficients *k_d_* were calculated from PAR profiles measured in 2011 along the transect T2 considering the depth range between 1.5 m and 10 m depth. The log-transformed PAR decreases linearly with depth and the slope is essentially constant suggesting that a constant light extinction coefficient is appropriate to describe PAR as function of depth. The mean of the light extinction coefficients obtained from all profiles measured in the transect from 9∶00 is *k_d_* = 0.56 m^−1^ (R^2^ = 0.99). This *k_d_* value was used to calculate PAR as function of depth in August 2011. The light intensity at the depth of the *P. rubescens* layer was determined from the incident light at the lake surface and the light extinction coefficient.

**Figure 9:**
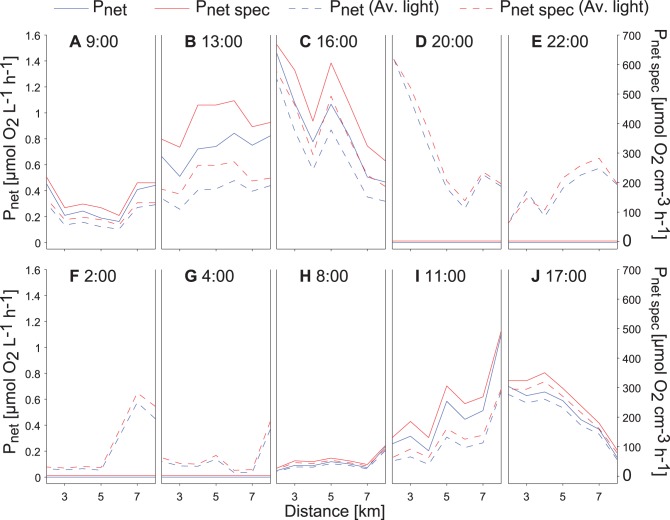
Spatiotemporal course O_2_ production rates. Specific net O_2_-production rate (*P_net spec_*) and net O_2_ production rate (*P_net_*) of *P. rubescens* at the depth of the maximum *P. rubescens* concentration during the 34-hour campaign (between 13^th^ and 14^th^ of August 2011). Calculations are based on the temporal course of clear sky radiation (solid lines) and on the 34-hour average clear sky radiation (dashed lines).

For the 34-hour campaign clear sky radiation was employed as incident light intensity. Using the average incident PAR at the lake surface over the 34-hour campaign (PAR_av_ = 219 µE m^−2^ s^−1^) *P_net spec_* of *P. rubescens* at the depth of the *P. rubescens* layer varies considerably along the transects (Fig. 9, dashed red lines). The differences in *P_net spec_* within transects can reach up to a factor of 9_._ (e.g. within transect D or transect F, Fig. 9). These differences illustrate the potential effect size of internal wave motion on the horizontal differences in the *P_net spec_* by *P. rubescens*. The horizontal variability of *P_net_* within transects (up to 10 times difference between the northernmost and southernmost stations within transect D and F) is very similar and parallel to that of *P_net spec_* (Fig. 9, dashed red lines).

Considering as incident light the daily course of PAR at the lake surface instead of the average PAR results in a significant change in *P_net_* by *P. rubescens*, because there is no production during night but increased production during the day time (Fig. 9, solid red lines). The transect mean of the *P_net spec_* at the depths of the *P. rubescens* layer shows a daily variation that is parallel to that of incident PAR (correlation R = 0.83; p = 0.003). The average *P_net spec_* over all transects differs only slightly between the calculations based on PAR_av_ (179 µmol O_2_ cm^−3^ h^−1^) and the daily course of PAR (161 µmol O_2_ cm^−3^ h^−1^). However, the daily mean horizontal difference in production differs because the horizontal differences in vertical displacement were particularly large at night (e.g. Fig. 5E) and no *P_net_* occurred when considering the daily course of PAR. Clearly, the horizontal variability in *P_net_* within the transects does not only depend on the variations in the depth of the *P. rubescens* layer along the transects but also on the timing between the patterns of layer displacement and the daily course of incident PAR.

Consequences of synchronisation between vertical displacements of *P. rubescen*s and the daily cycle in PAR for the spatial differences in *P_net spec_* of *P. rubescen*s are illustrated below for time periods beyond the 34-hour campaign. As profiles of the abundance of *P rubescen*s were measured only sporadically, the depth of the *P. rubescens* layer was estimated from temperature data assuming that the *P. rubescens* layer remains associated with the same temperatures. First, temperature at the depth of the maximum *P. rubescens* concentration, *T_P.rub_*, was determined using the MFP profiles from 10^th^ of August, 19^th^ of August and 21^st^ of September and *T_P.rub_* was linearly interpolated over time. Second, we calculated the depth *D_P.rub_*, at which the temperature in the linearly interpolated temperature profiles measured with the thermistor chains agrees with *T_P.rub_*. This procedure provides time series of *D_P.rub_* at the stations M1 and M2, respectively, which can be considered to represent time series of the depth of the *P. rubescens* layer at these stations. Using the data on incident PAR we calculated time series of PAR at the depth of the *P. rubescens* layer, i.e. *D_P.rub_*, at stations M1 and M2. These light intensities were employed to determine time series of O_2_-production rates of *P. rubescens* at stations M1 and M2.

**Figure 10:**
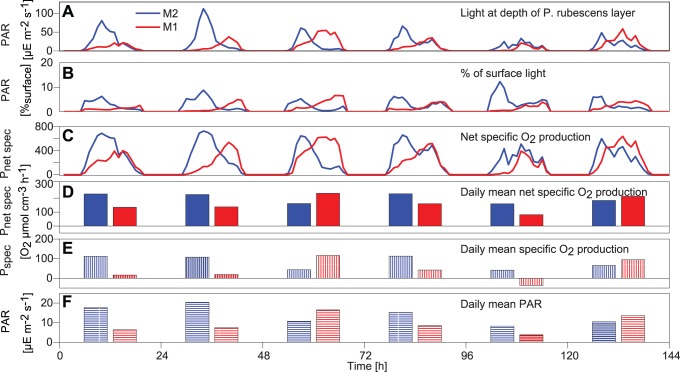
Time series of underwater light and O_2_-production rates of *P. rubescens* calculated at the depth of the *P. rubescens* layer between the 11^th^ and the 16^th^ of August 2011 at stations M1 (red) and M2 (blue). A: Amount of PAR available at the *P. rubescens* layer depth (µE = µmol photons). B: % PAR of surface radiation at the *P. rubescens* layer depth. C: Net O_2_-production rate (*P_net spec_*) at the *P. rubescens* layer depth. D: Daily mean net O_2_-production rate (*P_net spec_*) at the *P. rubescens* layer depth. E: Daily mean gross production rate (*P_spec_*) at *P. rubescens* layer depth F: Daily mean PAR at *P. rubescens* depth.

PAR at the depth of the *P. rubescens* layer changes with the time of the day because of the daily cycle of incident PAR and additionally varies because of the variation in layer depth. At the same times PAR at the depth of the *P. rubescens* layer can differ substantially between the two stations M1 and M2 because of the difference in *D_P.rub_* at the stations (Fig. 10A). *P_net spec_* at the depth of the *P. rubescens* layer has a similar pattern as the underwater PAR (Fig. 10C), but is somewhat dampened at high PAR because of the nonlinear P/I curve of *P. rubescens*. Between 11^th^ and 16^th^ of August 2011, the average *P_net spec_* at stations M1 and M2 is significantly different (pairwise t-test: p = 0.02) and is approximately 25% larger at M2 than at M1. The average *P_spec_* is also significant different (pairwise t-test: p = 0.02) and 100% larger at station M2 compared to station M1. The daily mean available PAR at the *P. rubescens* layer and the daily mean *P_spec_* also differ between stations and are up to 2.8, respectively 7, times larger at M2 than at M1 (Fig. 10E and F).

However, for long time periods of more than half a month, e.g. from 2^nd^ to 19^th^ of September 2011, the average *P_net spec_ and P_spec_* of *P. rubescens* does not significantly differ between stations M1 and M2 (pairwise t-test: p = 0.47).

The impact of variability in cloud cover on the estimated production rates can be tested by using clear sky radiation instead of measured solar radiation in the calculations above. However, as above, *P_net spec_* by *P. rubescens* is significantly different between the M1 and M2 in a 6-day time period (11^th^ and 16^th^ of August 2011: pairwise t-test: p = 0.005) but does not differ significantly for longer time periods, e. g. for the 2^nd^ to 19^th^ of September 2011 (pairwise t-test: p = 0.88).

## Discussion

### The distribution of phytoplankton and internal waves

Diatoms are found in Lake Ammer during the entire year [29] and were the second most abundant phytoplankton group during our experiments in August 2009 and 2011. The main proportion of the diatom population is located in the well mixed epilimnion, where production and upward transport by turbulent diffusion compensate sedimentation losses. Below the mixed layer depth the diatom biomass sharply declines. Comparison between cell counts with a microscope and MFP vertical profiles showed that the diatom concentration in the transition zone between the epi- and metalimnion is slightly underestimated by the MFP. This is most likely caused by the spatial overlap of the diatoms with *P. rubescens*, which is much more abundant at this depth range. The spatial overlap of these phytoplankton groups makes a reliable estimation of the low diatom concentrations from fluorescence of only a few pigments rather difficult. Nevertheless, vertical profiles of the cell counts of diatoms indicate a similar vertical structure as the data from the MFP, confirming the vertical separation of diatoms and *P. rubescens* in all our transects.

*P. rubescens,* the most abundant phytoplankton species in Lake Ammer, has a maximum population density in the metalimnion which is consistent with observations from other lakes (e.g. Lake Zürich [6], Lake Bourget [16]). A persistent phytoplankton population in the metalimnion requires that the average light intensity in the metalimnion is sufficient that net production exceeds respiration. Hence, the ratio of the average euphotic depth (*z_eu_*) to the average depth of the mixed layer (*z_mix_*) must exceed 1, as is the case for *P. rubescens* in all the lakes mentioned above. During our experiments in August 2011 the average euphotic depth in Lake Ammer was *z_eu_* ∼7.5 m and the average depth of the mixed layer was *z_mix_* ∼6 m, providing an average ratio *z_eu_*/*z_mix_* ∼1.25. This suggests that the average light intensity below the mixed layer was sufficient to sustain a *P. rubescens* population in the metalimnion during our experiments. Note, however, that the euphotic depth changes during the daily light cycle and that the vertical position of the *P. rubescens* layer within the water column also changes at sub-daily time scales. The overall production of *P. rubescens* therefore may depend on the timing between the changes in the vertical position of the *P. rubescens* layer and the daily changes in the incident light intensity.

The vertical distributions of diatoms and of *P. rubescens* are strongly affected by advective transport of the water and in particular by internal wave motions (e.g. [6], [10], [13]). Internal waves are ubiquitous features in lakes and are also common in Lake Ammer. The main characteristics of internal waves in lakes are horizontal currents in opposite directions in different depth layers, e.g. in an upper less dense and a deeper more dense layer in case of a vertical mode 1 wave, resulting in a vertical displacement of the interface between the layers. Although mixing can be induced by internal wave motions (e.g.: [12], [38]), the primary transport process due to internal waves is advection. Advection does not alter the concentration within water parcels, but moves the water parcels and the dissolved and suspended matter within them to different locations. The re-distribution of the water masses and associated phytoplankton and dissolved substances, results locally in changes in the vertical distribution of phytoplankton without the requirement of turbulent mixing. Such vertical displacements due to internal waves are especially visible in case of phytoplankton forming a DCM, e.g. *P. rubescens* (this study, [6], [13]).

The most prominent internal waves observed during our measuring campaigns on Lake Ammer were longitudinal V1H1 basin-scale internal seiches with a period of ∼23 hours that were modified by the Coriolis effect, resulting in an internal front moving counter-clockwise around the basin. In close connection with the internal front also vertical mode 2 internal waves occurred at the northern and southern end of the basin. The re-distribution of epilimnetic and hypolimnetic waters by the basin-scale internal seiche induced vertical displacements of the metalimnion by several metres in opposite direction at the northern and southern end of Lake Ammer. This caused similarly large vertical displacements of *P. rubescens*, which formed a DCM in the metalimnion. As *P. rubescens* produces toxic microcystins, the periodic vertical displacements of the *P. rubescens* layer alters the locally available habitat for e.g. toxin sensitive sessile or motile organisms that are not displaced by the internal wave in the same manner as *P. rubescens*.

The internal wave motion also effects the vertical distribution of phytoplankton in the epilimnon. At the lake ends the re-distribution of epilimnetic waters leads to a change in the thickness of the epilimnion und thus also in the vertical extent of the diatom layer (e.g. Fig. 8). A further consequence of the wave induced re-distribution of the epilimnetic diatoms is that the water column integrated biomass of the diatoms differs along longitudinal transects although total diatom biomass and diatom concentrations within the epilimnion do not change substantially (Fig. 8). Thus a vertical mode 1 internal seiche may introduce horizontal gradients in water column integrated abundances of epilimnetic species implying fluctuating resources locally for non-motile grazers, such as mussels. A further potential impact of a local increase in epilimnion thickness might be a reduction in the vertically averaged specific production rate. The available light and thus the specific production rate at the bottom of the epilimnion at locations with a thick epilimnion is lower than at locations with a thin epilimnion. However, the effect of internal waves on the production of an epilimnetic phytoplankton species is most likely smaller than that on the production of a metalimnetic phytoplankton species forming a DCM. In the former case only a fraction of the epilimnetic phytoplankton population is shifted to lower light intensities, whereas in the latter case internal waves cause a displacement of the entire population within the light gradient.

Because a vertical mode 1 internal seiche does not cause a spreading of the metalimnion, no substantial consequences are expected for the local water column abundance of metalimnetic species such as *P. rubescens*. However, vertical mode 2 internal waves are associated with a local increase in the thickness of the metalimnion, which results in a local increase in the water column integrated biomass of *P. rubescens* (e.g. Figs. 5F and 6).

In 2009, the basin-scale tilt of the isotherms suggests a basin-scale vertical 1 mode internal seiche. The spreading of the isotherms at the northern end of the lake suggests the simultaneous occurrence of a vertical mode 2 internal wave. The depth of the upper boundary of the metalimnion remains essentially the same along the longitudinal transect and hence the thickness of the epilimnion does as well. Consistently, water column abundance of diatoms is rather constant along the entire transect. In contrast, water column abundance of *P. rubescens* is largest towards the northern end of the lake. The horizontally displaced metalimnetic water containing *P. rubescens* is distributed towards the north resulting in a thicker metalimnion which contains the horizontally displaced *P. rubescens*.

Our conclusions on the connection between internal wave motions and phytoplankton distributions and water column biomass based on field data from Lake Ammer are consistent with conclusions based on numerical simulations of the conditions in Lac du Bourget by Cuypers et al. [6]. According to Cuypers et al. [6], V1H1-seiche motions cause substantial vertical displacements but only small variations in water column biomass of metalimnetic *P. rubescens.* Further, the V1H1-seiche causes changes in epilimnion thickness in Lac du Bourget, which may result in changes in water column biomass of epilimnetic green algae by ±50% [6]. Cuypers et al. [6] suggested that horizontal differences in the water column biomass of *P. rubescens* observed between the lake ends in Lac du Bourget may be explained by a V2H1-seiche, which, however, has a period of several days. Temperature and current time series from Lake Ammer (Fig. 2, 4) support the hypothesis that the vertical spreading of the metalimnion and the associated vertical spreading of the *P. rubescens* layer result from a second vertical-mode internal wave. The data from Lake Ammer further exemplify that a second vertical-mode internal wave indeed can generate horizontal differences in the water column biomass of *P. rubescens* (Fig. 5). However, the vertical spreading of the metalimnion occurs more often than expected for the V2H1-seiche and appears to be associated with the reflection of the front of the basin-scale Kelvin wave.

*P. rubescens* has the ability to regulate its buoyancy to avoid sedimentation losses and could potentially regulate against the displacements by internal waves. *P. rubescens* can adjust its overall density by regulating the volume of gas vesicles [39]. However, the production of these gas vesicles and hence also the regulation of buoyancy is a rather slow process and can induce vertical movements of typically less than 1 m d^−1^ [40]. The capacity of *P. rubescens* to regulate its buoyancy also depends on its primary production. During high rates of primary production carbohydrates are produced that increase the density of the *P. rubescens* cells and reduce its buoyancy [39]. Hence, upward movement of *P. rubescens* towards regions with increased light intensity may enhance primary production and thereby reduce the potential of buoyancy control of *P. rubescens*. In any case, the observed vertical displacements of the *P. rubescens layer* by the basin-scale internal seiche in Lake Ammer are ∼10 m within ∼12 hours and thus exceed the ability of active buoyancy regulation of *P. rubescens* by more than an order of magnitude. *P. rubescens* can therefore not avoid the internal wave induced passive motions in Lake Ammer by active movements.

The internal front associated with the Kelvin wave in Lake Ammer caused a very rapid vertical displacement of the metalimnion and thus also of the *P. rubescens* layer by 6 m within 2 hours (a speed of more than 70 m d^−1^) at the northern end of the transect at 20∶00 and at 22∶00 on 13 August 2011 (Fig. 5D and E). Such rapid displacements are associated with rapid pressure changes on the vesicles of *P. rubescens*. However, *P. rubescens* has a high tolerance to changing pressure [40], [41] such that the observed pressure fluctuations seem to not be lethal or cause a collapse of the gas vesicles.

Internal wave motions clearly affect the horizontal and vertical distribution of plankton (Figs. 5, 6, 8; see also [10], [13]), which complicates the interpretation of distributions of plankton or of other water constituents with respect to production, consumption and species interaction within the water column. For example, the time series of weekly or bi-weekly abundances of plankton measured in water samples from fixed depths in Lake Constance show substantial periodic variations that which may reflect the effect of internal wave motions rather than growth or interactions between species [42]. Similarly, horizontal distributions of phytoplankton assessed with remote sensing may be influenced by internal motions moving deep chlorophyll layers closer to the surface into the sensor range of the satellite, erroneously suggesting horizontal variations in water column phytoplankton abundance [43]. Our data illustrate that individual vertical profiles of phytoplankton may not provide reliable estimates of the mean depth of a DCM (here caused by *P. rubescens)*, nor of the mean vertical extend of the DCM or a near-surface diatom layer (Figs. 5, 6, 8). Further, much of the horizontal variability in the phytoplankton concentration may not result from biological processes but from vertical displacements due to interval waves (Figs. 5–8). However, if the temperature distribution has been measured together with the distributions of plankton and dissolved substances, temperature can be utilised to distinguish the effects of vertical displacements due to internal waves from effects of other processes (e.g. sedimentation, vertical migration, vertical mixing) on the three dimensional distributions of phytoplankton or dissolved substances. Vertical displacements of water masses are associated with the vertical displacement of all constituents and properties of the water, such as dissolved substances, suspended particles and organisms, and heat. In temperature stratified systems temperature can therefore be utilised as a tracer indicating the extent of the vertical displacements and is typically employed to characterise internal wave motions. In the absence of mixing, the vertical displacements of dissolved substances, neutrally buoyant suspended particles and organisms are exactly the same as the vertical displacements of temperature. As long as vertical temperature profiles are monotonic at each location, distributions of, e.g., particles, organisms or dissolved substances can be uniquely represented in a coordinate system that uses Euclidean coordinates in the horizontal dimension but replaces the vertical coordinate depth with the coordinate temperature. In this coordinate system, horizontal and temporal variations due to vertical advective displacements are not visible, because concentrations follow passively the displacement of the isotherms.

**Figure 11:**
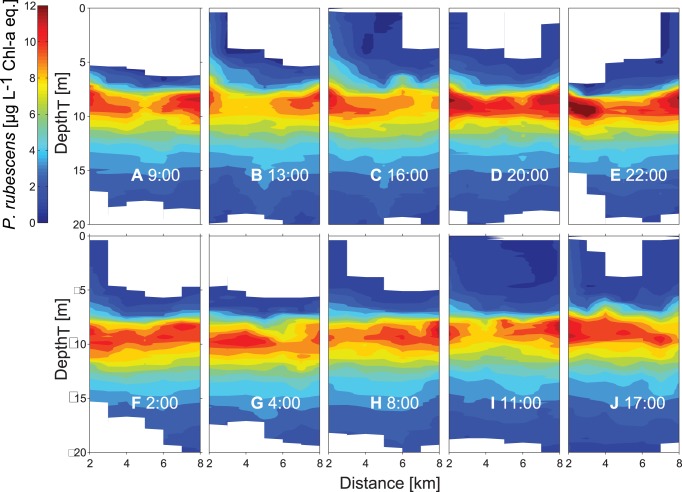
Spatiotemporal course of the *P. rubescens* layer after application of the temperature transformation. Concentrations of *P. rubescens* as function of depth_T_ are depicted along the transect T2 measured several times during the 34 h campaign from 13^th^ and 14^th^ of August 2011.

As it is uncommon to display vertical profiles and distributions in temperature space, distributions as function of temperature were transformed to distributions as function of a vertical spatial coordinate depth_T_ using an average temperature versus depth relationship that approximately corresponds to the undisturbed temperature stratification in the absence of vertical advective motions presuming that temperature is horizontally homogeneous in the undisturbed state. This transformation was applied to all transects of the 34 hours campaign, which removed most variations in the *P. rubescens* layer depth in all measured transects (Fig. 11). The depth and the vertical extent of the *P. rubescens* layer were nearly the same and the spatial heterogeneity was much lower in each transect (Fig. 11). In the temperature space transformed coordinate system, the *P. rubescens* layer was essentially horizontal and located at the same depth_T_ in all transects (Fig. 11), despite the passage of the internal front (Figs. 5E and 7E), the vertical mode 2 internal wave (Figs. 5F and 7F) and the vertical mode 1 internal seiche. These observations support the conclusion that *P. rubescens* does not perform substantial active movements in temperature space, i.e. relative to isotherms, suggesting that it essentially keeps its buoyancy independent of vertical displacements due to internal wave motions.

### Implications of internal waves for phytoplankton production rate

The re-distribution of phytoplankton due to internal waves not only affects habitat space for toxin-sensitive organisms and resource availability for higher trophic levels but also phytoplankton production [10]. Vertical displacements of phytoplankton species within the water column affect PAR available for production, because light intensity decreases with water depth. In the case of *P. rubescens*, the maximum growth rate is reached at comparatively low PAR-values (>10 µE m^−2^ s^−1^). Growth inhibition by *P. rubescens* does not occur for light levels below 200 µE m^−2^ s^−1^ [41]. Therefore, vertical displacements only affect the growth of *P. rubescens*, if light intensities fall below 10 µE m^−2^ s^−1^. However, as *P. rubescens* typically forms a DCM where light intensities are low, vertical displacements of *P. rubescens* affect the production of *P. rubescens*. [13]. As seiches cause periodic vertical displacements the increased production during one phase of the seiche is followed by a decrease in production during another phase. Because the effects of internal waves on phytoplankton production are largest, if high incident light intensity and wave crest are closely aligned [10], shifting the phase of an internal wave in relation to the daily light cycle, can modify the overall consequences of internal wave motions for primary production [10].

Considering vertical displacements of phytoplankton in a mean light gradient the long-term average production may differ from the production at the mean depth [10], because of the non-linear decrease in light intensity with depth which however is moderated by the non-linear increase in production with light intensity. The difference between mean production and production at mean depth depends on the amplitude of the vertical displacements, the light intensity at the mean depth of the DCM and the P/I-curve of the phytoplankton species. In the case of *P. rubescens* in Lake Ammer a sinusoidal oscillation with 10 m vertical peak to peak displacements around a mean depth of 10 m leads to an average *P_net spec_* of 133 µmol cm^−3^ h^−1^ which is 200% larger than the *P_net spec_* at 10 m depth (considering a light extinction coefficient of 0.56 m^−1^ and an incident light intensity of 457 µE m^−2^ s^−1^ corresponding to the daily mean average in August).

In addition, the vertical displacements due to the internal wave motions lead to substantial horizontal differences in the *P_net spec_* and *P_net_* of *P. rubescens* by up to a factor of 10 between the northern end and the centre of Lake Ammer (Fig. 9C and I). Compared with the variation in light intensity at the depth of the *P. rubescens* layer resulting from vertical displacements due to the internal waves, horizontal differences in the abundance of *P. rubescens* are only of minor importance for the horizontal differences in *P_net_* along the transects, as is indicated by the close agreement of the horizontal variability between *P_net spec_* and *P_net_* (Fig. 9, red and blue lines of same type).

*P_net__spec_* and *P_spec_* of *P. rubescens* can differ substantially between lake ends not only at specific times of the day but also on a daily average by up to 2 and 7 times, respectively (Figure 10C and D). These findings confirm recent observations from Lake Zürich which indicate that the daily mean production of *P. rubescens* is substantially affected by vertical displacements of *P. rubescens* within the water column [13]. However, Garneau et al. [13] measured 5 times within a 4 day time period along a transversal transect in Lake Zürich, which made it rather difficult to relate the observed differences in production to internal wave motions. In Lake Ammer measurements were therefore collected at opposite lake ends in a lengthwise direction and detailed information on the seiche motion were made available by collecting time series of temperature and current data at a high temporal resolution. These data support that the differences in the production of *P. rubescens* between the lake ends in Lake Ammer result from a basin-scale V1H1 internal seiche and that the horizontal differences in the vertical spreading of the *P. rubescens* layer result from vertical mode 2 internal waves. The production rates *P_net__spec_* and *P_spec_* not only differed between lake ends on a daily average but also at longer time scales. *P_spec_* averaged over 6 days was about twice as large at the southern as at the northern end of Lake Ammer. The long-term horizontal differences in *P_net__spec_* and *P_spec_* depend on the synchronization between daily light cycle and internal wave motion. In Lake Ammer, the period of the V1H1 mode longitudinal basin-scale internal seiche (∼23 hours) is only slightly smaller than the period of the daily light cycle. Hence, the daily course of the vertical displacement of the *P. rubescens* layer due to the internal seiche and the daily course of incident light may remain correlated at one end and anti-correlated at the other end of the basin for several days. In this case, the available light intensity at the depth of the *P. rubescens* layer is elevated at one end and reduced at the other end of the lake for several days depending on the phase of the internal seiche in relation to the daily light cycle. This explains the difference in average *P_net__spec_* and *P_spec_* of *P. rubescens* between stations M1 and M2 in Lake Ammer for the time period from the 11^th^ to the 16^th^ of August (Fig. 10).

During the 34 hours campaign, however, potential differences in *P_net__spec_* and *P_net_* due to the internal seiche were less effective, since the time periods with the largest vertical displacements of the *P. rubescens* layer along T2 occurred at night (Fig. 9D and E). However, this is not necessarily always the case because the period of the V1H1 internal seiche is slightly shorter than that of the daily light cycle. Hence, the internal front associated with the fundamental seiche may be slowly shifted towards daytime if the period of the internal seiche remains persistent for several days. The differences in underwater light availability and O_2_ production rate of *P. rubescens* at the opposite ends of the lake (stations M1 and M2) maintained over six days, but averaged out during the 18-day time period between the 2^nd^ and 19^th^ of September. How long horizontal differences in *P_net__spec_* and *P_spec_* persist depends on the period of the internal seiche and on the synchronization between internal seiche and daily light cycle. The differences in average O_2_ production rate at opposite lake ends may be sustained over several days if the internal seiches have periods of several days (e.g. 4 days in Lake Geneva [36]), but diminish very rapidly if internal seiches have periods of only a few hours (e.g. 3.7 hours in Lake Lunz, [36]). However, internal seiches with periods of about 24 hours can also lead to long-term horizontal differences in the production of *P. rubescens* as is demonstrated here for Lake Ammer. Daily winds forcing the phase of basin-scale internal waves may also support long-term horizontal gradients in phytoplankton production.

The timing of cloud cover in relation to the phase of daily cycle in light intensity and the vertical displacements due to internal seiches may additionally alter O_2_ production [10]. Cloud cover substantially reduced daily mean specific production by *P. rubescens* in Lake Ammer e.g. on 15 August 2011 (Figure 10E). Especially in the morning of this day cloud cover decreased the contrast in available light between the lake ends at the depth of the *P. rubescens* layer (Figure 10B and C). The differences in available light intensity between the lake ends caused by vertical displacements of *P. rubescens* (Figure 10B) are larger than those caused by the combined effect of cloud cover, vertical displacement and the daily cycle in light intensity (Figure 10A). However, as long as cloud cover is not synchronised with the seiche motion it does not substantially alter long-term averages of the relative proportion of *P_net spec_* between the lake ends. During the 6-day period from 11^th^ of August to 16^th^ of August average *P_net spec_* at M2 is 25% higher than at M1 independent of whether *P_net spec_* is calculated with the measured incident light that includes cloud cover or assuming clear sky conditions. Nevertheless, cloud cover leads to lower *P_net spec_* and therefore to a reduction of the absolute difference in the average *P_net spec_*. Considering clear sky conditions the absolute difference in *P_net spec_* between M1 and M2 averaged from 11^th^ of August to 16^th^ of August is 35% higher than the difference calculated from the measured incident light intensity that includes cloud cover.

Despite the temporal variations in cloud cover, the differences in the average *P_net spec_* between the opposite ends of Lake Ammer remained significantly different over the 6-day period shown in Figure 10.

The O_2_ production rate of *P. rubescens* was calculated under the assumption of no nutrient limitation. During the investigations period the SRP concentrations in the epi-, meta-, and hypolimnion were always ∼5 µg L^−1^ suggesting that phosphorus limitations were not severe. Even if nutrient limitation slightly reduces production rates compared with the estimates based on the formula of Walsby et al. [37], the differences in production rate between the northern and southern end of the lake still remain as they result from differences in the available light at the depth of the *P rubescens* layer.

In conclusion, the re-distribution of the phytoplankton by internal waves alters the resources cause horizontal differences locally the water column abundance especially of epilimnetic phytoplankton whereas vertical mode 2 internal waves most strongly influences the horizontal distribution of the water column abundance of phytoplankton forming a DCM in the metalimnion. Re-distribution of the toxin-producing *P. rubescens* by vertical mode 1 basin-scale internal waves affects the available niche space for toxin-sensitive organisms and in particular the available depth range for toxin-sensitive sessile organisms near the ends of the lake, but may potentially also alter the swimming behavior of fish. The vertical displacements due to internal wave motions additionally affect the production of phytoplankton, because of the decrease in light intensity with depth. Long-term horizontal differences in phytoplankton production, potentially contributing to phytoplankton patchiness, may be generated by basin-scale internal seiches depending on the period and synchronization of the displacements due to the internal waves with the daily cycle of incident light and on the phytoplankton growth rate. Fast-growing species may show more pronounced spatial differences in the abundance compared with slow growing-species.
